# Supplementary material for: End Sequence Analysis Toolkit (ESAT) expands the extractable information from single-cell RNA-seq data
Source: Genome Res. 2016 Oct;26(10):1397–410. doi: 10.1101/gr.207902.116 (PMC5052061; doi:10.1101/gr.207902.116)
Supplement: Supplemental Material [file supp_gr.207902.116_Supp_Materials.tar.gz › ESAT-esat_v0.1/doc/html/classbroad_1_1core_1_1sequence_1_1_sequence.html]

ESAT: broad.core.sequence.Sequence Class Reference


ESAT


- Main Page
- Related Pages
- Packages
- Classes
- Files

- Class List
- Class Index
- Class Hierarchy
- Class Members

All Classes Namespaces Files Functions Variables Enumerator Pages

- broad
- core
- sequence
- Sequence

Classes |
Public Member Functions |
Static Public Member Functions |
Static Public Attributes |
List of all members

broad.core.sequence.Sequence Class Reference

Inheritance diagram for broad.core.sequence.Sequence:

[legend]

Collaboration diagram for broad.core.sequence.Sequence:

[legend]

|  |  |
| --- | --- |
| Classes | |
| class | **KmerInfo** |
|  | |

|  |  |
| --- | --- |
| Public Member Functions | |
|  | Sequence (String id) |
|  | |
|  | Sequence (String id, boolean isLarge) |
|  | |
|  | Sequence (String id, int expectedSize) |
|  | |
| String | getId () |
|  | |
| void | setForwardStrand (boolean isForwardStrand) |
|  | |
| void | setId (String id) |
|  | |
| void | unloadSequence () |
|  | |
| void | unloadEncodedSequence () |
|  | |
| void | unloadAllSequences () |
|  | |
| int | getLength () |
|  | |
| void | setCharAt (int position, char newCharacter) throws IllegalAccessException |
|  | |
| String | getSequenceBases () |
|  | |
| void | setSequenceBases (String sequence) |
|  | |
| void | append (String partialSequence) |
|  | |
| Sequence | shuffle () |
|  | |
| void | append (char c) |
|  | |
| boolean | isGap (int position) |
|  | |
| List< GenomicAnnotation > | getSoftmaskedRegions () |
|  | |
| int | countSoftMaskedBases () |
|  | |
| void | maskSoftmaskedRegions () |
|  | |
| short[] | getEncodedSequence () |
|  | |
| Matrix | getVectorEncodedSequence () |
|  | |
| Matrix | encodeSequenceAsVector () |
|  | |
| short[] | encodeSequence () |
|  | |
| short[] | encodeSequenceIgnoreCase () |
|  | |
| short[] | encodeSequenceIgnoreCase (boolean distinguishMissingSequence) |
|  | |
| List< Short > | compute454Flow (char[] flowOrder) |
|  | |
| List< Short > | compute454Flow (char[] flowOrder, int start, int end) |
|  | |
| float | gcContent () |
|  | |
| boolean | contains (String motif) |
|  | |
| void | reverse () |
|  | |
| void | uppercase () |
|  | |
| void | mask (Annotation region, boolean softmask) |
|  | |
| SequenceRegion | getRegion (int start, int end) |
|  | |
| void | getRegion (SequenceRegion region) |
|  | |
| void | getRegion (SequenceRegion region, boolean softmask) |
|  | |
| void | getRegion (SequenceRegion region, boolean softmask, Map< String, IntervalTree< Alignments >> okRepeats) |
|  | |
| void | getRegions (List<?extends SequenceRegion > regions) |
|  | |
| SequenceRegion | extractRegionBasedOnGC (float targetGC, int size, int buffer) |
|  | |
| boolean | isForwardStrand () |
|  | |
| StringBuilder | getSequenceBuilder () |
|  | |
| int | getGapsSize () |
|  | |
| WindowSlider | getSlider (int windowSize, int overlap) |
|  | |
| List< SequenceRegion > | chunk (int chunkSize, int chunkOverlap) |
|  | |
| int | getEnd () |
|  | |
| List< int[]> | findUngappedSequenceChunks () |
|  | |
| Collection< Annotation > | getMatches (String s, int windowStart, int windowEnd, boolean includeRC) |
|  | |
| Sequence | getSubSequence (SequenceRegion region, int extension) |
|  | |
| Sequence | getSubSequence (String name, int start, int end, int extension) |
|  | |
| Sequence | getSubSequence (String name, int start, int end) |
|  | |
| Sequence | getSubsequence (Gene annot) |
|  | |
| Sequence | getSubsequence (Annotation annot) |
|  | |
| void | setCapacity (int size) |
|  | |
| Sequence | scramble () |
|  | |
| Sequence | getAntisense () |
|  | |
| String | getRefseqGeneName () |
|  | |
| String | getRefseqSpeciesName () |
|  | |
| String | getRefseqRnaClassName () |
|  | |
| Map< String, KmerInfo > | buildKmerMap (int kmer) |
|  | |

|  |  |
| --- | --- |
| Static Public Member Functions | |
| static short[] | encodeSequence (StringBuilder bases) |
|  | |
| static short[] | encodeSequenceIgnoreCase (StringBuilder sequence) |
|  | |
| static short[] | encodeSequenceIgnoreCase (StringBuilder sequence, boolean distinguishMissingSequence) |
|  | |
| static float | computeGCContent (String dnaString) |
|  | |
| static float[] | computeSequenceComposition (String dnaString) |
|  | |
| static Sequence | reverseSequence (Sequence seq) |
|  | |
| static String | reverseSequence (String seq) |
|  | |
| static String | decode (short[] shortEncodedSequence) |
|  | |
| static String | generateRandomSequence (int oligoSize) |
|  | |
| static String | get3Prime (String seq, int numBases) |
|  | |
| static Collection< String > | generateAll5Mers () |
|  | |
| static Collection< String > | generateAllKmers (int k) |
|  | |
| static String | complement (String seq) |
|  | |

|  |  |
| --- | --- |
| Static Public Attributes | |
| static final char[] | SHORT\_READ\_FLOW = {'T','A','C','G'} |
|  | |
| static final char[] | LONG\_READ\_FLOW = {'G','A','T','C'} |
|  | |
| static final short | SHORT\_ENCODED\_A = 0 |
|  | |
| static final short | SHORT\_ENCODED\_C = 1 |
|  | |
| static final short | SHORT\_ENCODED\_G = 2 |
|  | |
| static final short | SHORT\_ENCODED\_T = 3 |
|  | |
| static final short | SHORT\_ENCODED\_GAP = 4 |
|  | |
| static final short | SHORT\_ENCODED\_a = 5 |
|  | |
| static final short | SHORT\_ENCODED\_c = 6 |
|  | |
| static final short | SHORT\_ENCODED\_g = 7 |
|  | |
| static final short | SHORT\_ENCODED\_t = 8 |
|  | |
| static final short | SHORT\_ENCODED\_N = 9 |
|  | |

## Constructor & Destructor Documentation

|  |  |  |  |  |  |
| --- | --- | --- | --- | --- | --- |
| broad.core.sequence.Sequence.Sequence | ( | String | *id* | ) |  |

Here is the caller graph for this function:

|  |  |  |  |
| --- | --- | --- | --- |
| broad.core.sequence.Sequence.Sequence | ( | String | *id*, |
|  |  | boolean | *isLarge* |
|  | ) |  |  |

|  |  |  |  |
| --- | --- | --- | --- |
| broad.core.sequence.Sequence.Sequence | ( | String | *id*, |
|  |  | int | *expectedSize* |
|  | ) |  |  |

## Member Function Documentation

|  |  |  |  |  |  |
| --- | --- | --- | --- | --- | --- |
| void broad.core.sequence.Sequence.append | ( | String | *partialSequence* | ) |  |

Here is the caller graph for this function:

|  |  |  |  |  |  |
| --- | --- | --- | --- | --- | --- |
| void broad.core.sequence.Sequence.append | ( | char | *c* | ) |  |

|  |  |  |  |  |  |
| --- | --- | --- | --- | --- | --- |
| Map<String, KmerInfo> broad.core.sequence.Sequence.buildKmerMap | ( | int | *kmer* | ) |  |

Gvien a kmer size, it builds a map of all kmers found

Parameters
:   |  |  |
    | --- | --- |
    | kmer |  |

Here is the call graph for this function:

|  |  |  |  |
| --- | --- | --- | --- |
| List<SequenceRegion> broad.core.sequence.Sequence.chunk | ( | int | *chunkSize*, |
|  |  | int | *chunkOverlap* |
|  | ) |  |  |

Here is the call graph for this function:

|  |  |  |  |  |  |
| --- | --- | --- | --- | --- | --- |
| static String broad.core.sequence.Sequence.complement | ( | String | *seq* | ) |  |

static

Here is the caller graph for this function:

|  |  |  |  |  |  |
| --- | --- | --- | --- | --- | --- |
| List<Short> broad.core.sequence.Sequence.compute454Flow | ( | char[] | *flowOrder* | ) |  |

|  |  |  |  |
| --- | --- | --- | --- |
| List<Short> broad.core.sequence.Sequence.compute454Flow | ( | char[] | *flowOrder*, |
|  |  | int | *start*, |
|  |  | int | *end* |
|  | ) |  |  |

|  |  |  |  |  |  |
| --- | --- | --- | --- | --- | --- |
| static float broad.core.sequence.Sequence.computeGCContent | ( | String | *dnaString* | ) |  |

static

Here is the caller graph for this function:

|  |  |  |  |  |  |
| --- | --- | --- | --- | --- | --- |
| static float [] broad.core.sequence.Sequence.computeSequenceComposition | ( | String | *dnaString* | ) |  |

static

Here is the caller graph for this function:

|  |  |  |  |  |  |
| --- | --- | --- | --- | --- | --- |
| boolean broad.core.sequence.Sequence.contains | ( | String | *motif* | ) |  |

Here is the call graph for this function:

Here is the caller graph for this function:

|  |  |  |  |  |
| --- | --- | --- | --- | --- |
| int broad.core.sequence.Sequence.countSoftMaskedBases | ( |  | ) |  |

|  |  |  |  |  |  |
| --- | --- | --- | --- | --- | --- |
| static String broad.core.sequence.Sequence.decode | ( | short[] | *shortEncodedSequence* | ) |  |

static

|  |  |  |  |  |
| --- | --- | --- | --- | --- |
| short [] broad.core.sequence.Sequence.encodeSequence | ( |  | ) |  |

|  |  |  |  |  |  |
| --- | --- | --- | --- | --- | --- |
| static short [] broad.core.sequence.Sequence.encodeSequence | ( | StringBuilder | *bases* | ) |  |

static

|  |  |  |  |  |
| --- | --- | --- | --- | --- |
| Matrix broad.core.sequence.Sequence.encodeSequenceAsVector | ( |  | ) |  |

Here is the caller graph for this function:

|  |  |  |  |  |
| --- | --- | --- | --- | --- |
| short [] broad.core.sequence.Sequence.encodeSequenceIgnoreCase | ( |  | ) |  |

Here is the caller graph for this function:

|  |  |  |  |  |  |
| --- | --- | --- | --- | --- | --- |
| short [] broad.core.sequence.Sequence.encodeSequenceIgnoreCase | ( | boolean | *distinguishMissingSequence* | ) |  |

Here is the call graph for this function:

|  |  |  |  |  |  |
| --- | --- | --- | --- | --- | --- |
| static short [] broad.core.sequence.Sequence.encodeSequenceIgnoreCase | ( | StringBuilder | *sequence* | ) |  |

static

Here is the call graph for this function:

|  |  |  |  |
| --- | --- | --- | --- |
| static short [] broad.core.sequence.Sequence.encodeSequenceIgnoreCase | ( | StringBuilder | *sequence*, |
|  |  | boolean | *distinguishMissingSequence* |
|  | ) |  |  |

static

|  |  |  |  |
| --- | --- | --- | --- |
| SequenceRegion broad.core.sequence.Sequence.extractRegionBasedOnGC | ( | float | *targetGC*, |
|  |  | int | *size*, |
|  |  | int | *buffer* |
|  | ) |  |  |

Here is the call graph for this function:

|  |  |  |  |  |
| --- | --- | --- | --- | --- |
| List<int []> broad.core.sequence.Sequence.findUngappedSequenceChunks | ( |  | ) |  |

Finds the list of ungapped regions in the sequence.

Returns
:   A list of two sized integer arrays with the start and end of the ungapped regions. The convention here is semi closed intervals, each list items is of the form [start, end).

Here is the call graph for this function:

|  |  |  |  |  |
| --- | --- | --- | --- | --- |
| float broad.core.sequence.Sequence.gcContent | ( |  | ) |  |

Here is the call graph for this function:

|  |  |  |  |  |
| --- | --- | --- | --- | --- |
| static Collection<String> broad.core.sequence.Sequence.generateAll5Mers | ( |  | ) |  |

static

|  |  |  |  |  |  |
| --- | --- | --- | --- | --- | --- |
| static Collection<String> broad.core.sequence.Sequence.generateAllKmers | ( | int | *k* | ) |  |

static

Get all kmers composed of the bases A, C, G, T

Parameters
:   |  |  |
    | --- | --- |
    | k | K |

Returns
:   All 4^k kmers

|  |  |  |  |  |  |
| --- | --- | --- | --- | --- | --- |
| static String broad.core.sequence.Sequence.generateRandomSequence | ( | int | *oligoSize* | ) |  |

static

|  |  |  |  |
| --- | --- | --- | --- |
| static String broad.core.sequence.Sequence.get3Prime | ( | String | *seq*, |
|  |  | int | *numBases* |
|  | ) |  |  |

static

|  |  |  |  |  |
| --- | --- | --- | --- | --- |
| Sequence broad.core.sequence.Sequence.getAntisense | ( |  | ) |  |

Here is the call graph for this function:

|  |  |  |  |  |
| --- | --- | --- | --- | --- |
| short [] broad.core.sequence.Sequence.getEncodedSequence | ( |  | ) |  |

|  |  |  |  |  |
| --- | --- | --- | --- | --- |
| int broad.core.sequence.Sequence.getEnd | ( |  | ) |  |

Here is the call graph for this function:

|  |  |  |  |  |
| --- | --- | --- | --- | --- |
| int broad.core.sequence.Sequence.getGapsSize | ( |  | ) |  |

|  |  |  |  |  |
| --- | --- | --- | --- | --- |
| String broad.core.sequence.Sequence.getId | ( |  | ) |  |

Here is the caller graph for this function:

|  |  |  |  |  |
| --- | --- | --- | --- | --- |
| int broad.core.sequence.Sequence.getLength | ( |  | ) |  |

Here is the caller graph for this function:

|  |  |  |  |
| --- | --- | --- | --- |
| Collection<Annotation> broad.core.sequence.Sequence.getMatches | ( | String | *s*, |
|  |  | int | *windowStart*, |
|  |  | int | *windowEnd*, |
|  |  | boolean | *includeRC* |
|  | ) |  |  |

Get all matches for a string as annotations with respect to this sequence Search a window Case is ignored

Parameters
:   |  |  |
    | --- | --- |
    | s | The string |
    | windowStart | Start position of window to search |
    | windowEnd | End of window to search |
    | includeRC | Also search for reverse complement of string |

Returns
:   Collection of annotations representing string matches

Here is the call graph for this function:

|  |  |  |  |  |
| --- | --- | --- | --- | --- |
| String broad.core.sequence.Sequence.getRefseqGeneName | ( |  | ) |  |

Get the Refseq gene name which appears between the last pair of parentheses in fasta header

Returns
:   gene name

|  |  |  |  |  |
| --- | --- | --- | --- | --- |
| String broad.core.sequence.Sequence.getRefseqRnaClassName | ( |  | ) |  |

Here is the call graph for this function:

|  |  |  |  |  |
| --- | --- | --- | --- | --- |
| String broad.core.sequence.Sequence.getRefseqSpeciesName | ( |  | ) |  |

Get the species name that appears right after the transcript ID or the PREDICTED tag

Returns

Here is the call graph for this function:

|  |  |  |  |
| --- | --- | --- | --- |
| SequenceRegion broad.core.sequence.Sequence.getRegion | ( | int | *start*, |
|  |  | int | *end* |
|  | ) |  |  |

Parameters
:   |  |  |
    | --- | --- |
    | start | - start of the region to extract the starting base will be included starting at 0 |
    | end | - the end of the region to extract, the base at position end will not be included. |

Returns

Here is the call graph for this function:

Here is the caller graph for this function:

|  |  |  |  |  |  |
| --- | --- | --- | --- | --- | --- |
| void broad.core.sequence.Sequence.getRegion | ( | SequenceRegion | *region* | ) |  |

Here is the call graph for this function:

|  |  |  |  |
| --- | --- | --- | --- |
| void broad.core.sequence.Sequence.getRegion | ( | SequenceRegion | *region*, |
|  |  | boolean | *softmask* |
|  | ) |  |  |

Here is the call graph for this function:

|  |  |  |  |
| --- | --- | --- | --- |
| void broad.core.sequence.Sequence.getRegion | ( | SequenceRegion | *region*, |
|  |  | boolean | *softmask*, |
|  |  | Map< String, IntervalTree< Alignments >> | *okRepeats* |
|  | ) |  |  |

Here is the call graph for this function:

|  |  |  |  |  |  |
| --- | --- | --- | --- | --- | --- |
| void broad.core.sequence.Sequence.getRegions | ( | List<?extends SequenceRegion > | *regions* | ) |  |

Here is the call graph for this function:

|  |  |  |  |  |
| --- | --- | --- | --- | --- |
| String broad.core.sequence.Sequence.getSequenceBases | ( |  | ) |  |

Here is the caller graph for this function:

|  |  |  |  |  |
| --- | --- | --- | --- | --- |
| StringBuilder broad.core.sequence.Sequence.getSequenceBuilder | ( |  | ) |  |

Here is the call graph for this function:

|  |  |  |  |
| --- | --- | --- | --- |
| WindowSlider broad.core.sequence.Sequence.getSlider | ( | int | *windowSize*, |
|  |  | int | *overlap* |
|  | ) |  |  |

|  |  |  |  |  |
| --- | --- | --- | --- | --- |
| List<GenomicAnnotation> broad.core.sequence.Sequence.getSoftmaskedRegions | ( |  | ) |  |

Here is the call graph for this function:

|  |  |  |  |
| --- | --- | --- | --- |
| Sequence broad.core.sequence.Sequence.getSubSequence | ( | SequenceRegion | *region*, |
|  |  | int | *extension* |
|  | ) |  |  |

Here is the call graph for this function:

Here is the caller graph for this function:

|  |  |  |  |
| --- | --- | --- | --- |
| Sequence broad.core.sequence.Sequence.getSubSequence | ( | String | *name*, |
|  |  | int | *start*, |
|  |  | int | *end*, |
|  |  | int | *extension* |
|  | ) |  |  |

Get subsequence with extension

Parameters
:   |  |  |
    | --- | --- |
    | name | Name of new sequence to return |
    | start | Start position of subsequence |
    | end | Position after last position to include |
    | extension | Extension |

Returns
:   The subsequence

Here is the call graph for this function:

|  |  |  |  |
| --- | --- | --- | --- |
| Sequence broad.core.sequence.Sequence.getSubSequence | ( | String | *name*, |
|  |  | int | *start*, |
|  |  | int | *end* |
|  | ) |  |  |

Get subsequence

Parameters
:   |  |  |
    | --- | --- |
    | name | Name of new sequence to return |
    | start | Start position of subsequence |
    | end | Position after last position to include |

Returns
:   The subsequence

Here is the call graph for this function:

|  |  |  |  |  |  |
| --- | --- | --- | --- | --- | --- |
| Sequence broad.core.sequence.Sequence.getSubsequence | ( | Gene | *annot* | ) |  |

Get the spliced transcribed sequence of an annotation Bases are reported in 5' to 3' direction

Parameters
:   |  |  |
    | --- | --- |
    | annot | The annotation |

Returns
:   Sequence with same name as annotation containing the transcribed sequence

Here is the call graph for this function:

|  |  |  |  |  |  |
| --- | --- | --- | --- | --- | --- |
| Sequence broad.core.sequence.Sequence.getSubsequence | ( | Annotation | *annot* | ) |  |

Get the spliced transcribed sequence of an annotation Bases are reported in 5' to 3' direction

Parameters
:   |  |  |
    | --- | --- |
    | annot | The annotation |

Returns
:   Sequence with same name as annotation containing the transcribed sequence

Here is the call graph for this function:

|  |  |  |  |  |
| --- | --- | --- | --- | --- |
| Matrix broad.core.sequence.Sequence.getVectorEncodedSequence | ( |  | ) |  |

|  |  |  |  |  |
| --- | --- | --- | --- | --- |
| boolean broad.core.sequence.Sequence.isForwardStrand | ( |  | ) |  |

Here is the caller graph for this function:

|  |  |  |  |  |  |
| --- | --- | --- | --- | --- | --- |
| boolean broad.core.sequence.Sequence.isGap | ( | int | *position* | ) |  |

Here is the call graph for this function:

|  |  |  |  |
| --- | --- | --- | --- |
| void broad.core.sequence.Sequence.mask | ( | Annotation | *region*, |
|  |  | boolean | *softmask* |
|  | ) |  |  |

Mask part of sequence

Parameters
:   |  |  |
    | --- | --- |
    | region | Region to mask. Masks blocks only. |
    | softmask | If true, mask to lower case. If false, mask to Ns. |

Here is the call graph for this function:

Here is the caller graph for this function:

|  |  |  |  |  |
| --- | --- | --- | --- | --- |
| void broad.core.sequence.Sequence.maskSoftmaskedRegions | ( |  | ) |  |

|  |  |  |  |  |
| --- | --- | --- | --- | --- |
| void broad.core.sequence.Sequence.reverse | ( |  | ) |  |

Here is the call graph for this function:

|  |  |  |  |  |  |
| --- | --- | --- | --- | --- | --- |
| static Sequence broad.core.sequence.Sequence.reverseSequence | ( | Sequence | *seq* | ) |  |

static

Here is the call graph for this function:

Here is the caller graph for this function:

|  |  |  |  |  |  |
| --- | --- | --- | --- | --- | --- |
| static String broad.core.sequence.Sequence.reverseSequence | ( | String | *seq* | ) |  |

static

Here is the call graph for this function:

|  |  |  |  |  |
| --- | --- | --- | --- | --- |
| Sequence broad.core.sequence.Sequence.scramble | ( |  | ) |  |

Here is the call graph for this function:

|  |  |  |  |  |  |
| --- | --- | --- | --- | --- | --- |
| void broad.core.sequence.Sequence.setCapacity | ( | int | *size* | ) |  |

|  |  |  |  |
| --- | --- | --- | --- |
| void broad.core.sequence.Sequence.setCharAt | ( | int | *position*, |
|  |  | char | *newCharacter* |
|  | ) |  | throws IllegalAccessException |

|  |  |  |  |  |  |
| --- | --- | --- | --- | --- | --- |
| void broad.core.sequence.Sequence.setForwardStrand | ( | boolean | *isForwardStrand* | ) |  |

Here is the call graph for this function:

|  |  |  |  |  |  |
| --- | --- | --- | --- | --- | --- |
| void broad.core.sequence.Sequence.setId | ( | String | *id* | ) |  |

|  |  |  |  |  |  |
| --- | --- | --- | --- | --- | --- |
| void broad.core.sequence.Sequence.setSequenceBases | ( | String | *sequence* | ) |  |

|  |  |  |  |  |
| --- | --- | --- | --- | --- |
| Sequence broad.core.sequence.Sequence.shuffle | ( |  | ) |  |

Here is the call graph for this function:

|  |  |  |  |  |
| --- | --- | --- | --- | --- |
| void broad.core.sequence.Sequence.unloadAllSequences | ( |  | ) |  |

Here is the call graph for this function:

|  |  |  |  |  |
| --- | --- | --- | --- | --- |
| void broad.core.sequence.Sequence.unloadEncodedSequence | ( |  | ) |  |

Here is the caller graph for this function:

|  |  |  |  |  |
| --- | --- | --- | --- | --- |
| void broad.core.sequence.Sequence.unloadSequence | ( |  | ) |  |

Here is the caller graph for this function:

|  |  |  |  |  |
| --- | --- | --- | --- | --- |
| void broad.core.sequence.Sequence.uppercase | ( |  | ) |  |

## Member Data Documentation

final char [] broad.core.sequence.Sequence.LONG\_READ\_FLOW = {'G','A','T','C'}

static

final short broad.core.sequence.Sequence.SHORT\_ENCODED\_A = 0

static

final short broad.core.sequence.Sequence.SHORT\_ENCODED\_a = 5

static

final short broad.core.sequence.Sequence.SHORT\_ENCODED\_C = 1

static

final short broad.core.sequence.Sequence.SHORT\_ENCODED\_c = 6

static

final short broad.core.sequence.Sequence.SHORT\_ENCODED\_G = 2

static

final short broad.core.sequence.Sequence.SHORT\_ENCODED\_g = 7

static

final short broad.core.sequence.Sequence.SHORT\_ENCODED\_GAP = 4

static

final short broad.core.sequence.Sequence.SHORT\_ENCODED\_N = 9

static

final short broad.core.sequence.Sequence.SHORT\_ENCODED\_T = 3

static

final short broad.core.sequence.Sequence.SHORT\_ENCODED\_t = 8

static

final char [] broad.core.sequence.Sequence.SHORT\_READ\_FLOW = {'T','A','C','G'}

static

---

The documentation for this class was generated from the following file:

- java/broad/core/sequence/Sequence.java


---

Generated on Fri Aug 15 2014 15:10:56 for ESAT by  

 1.8.7
